# Corneal Aberrations, Contrast Sensitivity, and Light Distortion in Orthokeratology Patients: 1-Year Results

**DOI:** 10.1155/2016/8453462

**Published:** 2016-10-27

**Authors:** Elena Santolaria-Sanz, Alejandro Cerviño, José. M. González-Méijome

**Affiliations:** ^1^Private Practice, Onda, Castellon, Spain; ^2^Optometry Research Group, Department of Optics & Optometry & Vision Sciences, Universidad de Valencia, Valencia, Spain; ^3^CEORLab-Center of Physics (Optometry), Universidade do Minho, Braga, Portugal

## Abstract

*Purpose.* To evaluate the corneal higher-order aberrations (HOA), contrast sensitivity function (CSF), and light distortion (LD) in patients undergoing orthokeratology (OK).* Methods.* Twenty healthy subjects (mean age: 21.40 ± 8 years) with mean spherical equivalent refractive error M = −2.19 ± 0.97 D were evaluated at 1 day, 1 month, and 1 year after starting OK treatment. Monocular LD, photopic monocular CSF, and corneal HOA for 6 mm pupil size were measured.* Results.* LD showed an increase after the first night (*p* < 0.05) and recovery to baseline after 1 month, remaining stable after 1 year (*p* > 0.05). Spherical-like, coma-like, and secondary astigmatism HOA RMS increased significantly (*p* ≤ 0.022) from baseline to 1-month visit, remaining unchanged over the follow-up. Contrast sensitivity for medium frequencies (3.0, 4.24, and 6.00 cpd) was significantly correlated with LD parameters at baseline (*r* ≤ −0.529, *p* < 0.001). However, after 1 year of treatment, this correlation was only statistically significant for 12 cpd spatial frequency (*r* ≤ −0.565, *p* < 0.001). Spherical-like RMS for 6 mm pupil size correlated with irregularity of the LD (*r* = −0.420, *p* < 0.05) at the 1-year visit.* Conclusion.* LD experienced by OK patients recovers after one month of treatment and remains stable in the long term while optical aberrations remain significantly higher than baseline.

## 1. Introduction

Orthokeratology (OK) provides independence of conventional compensation by spectacles or contact lenses during the waking hours [1]. For myopia correction central cornea is flattened to achieve the desired reduction in the power of the anterior corneal surface, while the midperipheral cornea steepens [2, 3] as a result of the epithelial thickness redistribution from the center [4]. This treatment deteriorates the quality of vision [5–8] and it has been shown that such changes are dependent on pupil size [9] and strongly affected by the amount of refractive error being corrected [10].

In clinical practice, OK subjects usually complain of subjective perception of dysphotopic phenomena in the form of haloes, ghosting, or glare (here considered as light distortion phenomena or LD). Those are more intense at the beginning of treatment and decrease over time [11, 12]. We have observed in a cross-sectional study that OK subjects report subjective complaints that are transient during the first days or weeks of treatment. Common complaints include perception of haloes and starburst around light sources, either in outdoor (i.e., car lights) or indoor conditions [13]. In a different study, we have quantified the amount of LD over the period of adaptation to OK treatment in a cohort of 29 subjects and observed that LD phenomena increase at the first day but decrease again towards baseline values after 30 days [14]. However, in that study, we were not able to measure LD phenomena beyond the first month. Thus, we cannot ensure that the adaptation period does not undergo further changes in the long term. Other authors found a correlation between corneal irregularity and asymmetry parameters after 1 month of OK treatment [15], what might suggest that the optical quality of the front corneal surface during OK treatment might affect LD phenomena. Considering that our previous study showed that LD improves from 1 day to 1 month of treatment despite the increase in HOA up to 7 days after initiation of treatment, becoming stable thereafter, it has been hypothesized that LD is not related to HOA themselves or that there is an LD adaptation process even when the optical quality of the anterior corneal surface remains significantly affected by spherical-like and coma-like HOA [2, 3]. While several studies have addressed the longitudinal changes in HOA and contrast sensitivity (CS) over a period of 1 year, to date no study has addressed the temporal changes in LD phenomena beyond 1 month of treatment.

Different devices are available for measuring the disturbances surrounding bright spots against dark background; some of them use software [16–20]; others use custom-made [21] or commercially available ones [19]. We use a physical display to present the stimuli as this allows us to have a wider dynamic range of luminance from the main glare source and the detection peripheral stimuli [20] and this instrument has been shown to be consistent with [17] and sensitive to changes in higher-order aberrations (HOA) artificially induced [18].

Thus, the purpose of the present study was to evaluate the long-term visual effects of corneal optical quality degradation in OK patients by measuring the LD phenomena and CSF.

## 2. Methods

A total of 20 neophyte subjects were recruited and fitted with OK lenses for myopia correction. Subjects were followed and wore their lenses successfully for at least 1 year. Demographic and refractive data of subjects are presented in Table 1. Inclusion criteria required that they were over 18 years of age, had less than 1.00 diopters (D) of refractive astigmatism, were free of ocular disease, had not contraindication for overnight CL wear, and presented a best corrected monocular visual acuity of 0.90 decimals (20/25) or better.

Subjects were informed of the purpose of the study and signed a consent form after all of their questions had been answered. Following the tenets of the Declaration of Helsinki, the protocol of the study has been reviewed and approved by the IRB. Subjects underwent a comprehensive optometric examination.

### 2.1. Outcome Measures

In this study, all measures have been obtained monocularly in order to correlate aberration data with clinical data obtained with the different methodologies. Subjective baseline refraction and refraction at the time of data collection were determined as the spherocylindrical combination. Endpoint criterion was the highest positive (or less negative) refraction that allowed the patient to achieve their maximum visual acuity, in agreement with usual refraction procedures to control for accommodation effects.

LD was analyzed with an experimental prototype [20] at a distance of 2.0 m in a darkened room. It consists of an array of 240 1 mm wide LEDs distributed radially at 15° intervals over 160 mm, with a linear separation of 10 mm around a central 5 mm white LED that acts as glare source, as previously described [17, 18, 20]. The system is controlled by a custom-made software that interfaces with the patient to detect the peripheral stimuli seen and discriminate them from those hidden by the central glare source. Characteristics and examination procedures in the context of assessment of OK patients have been previously described [14]. In brief, in totally darkened room, the instrument presents the central source of glare at maximum intensity while the peripheral LEDs are randomly turned on and off. The patient provides feedback regarding the stimuli that can be seen by clicking a remote actuator. The system reads and stores the feedback information and within 45 to 75 seconds the instrument provides a drawing of the area where the peripheral stimuli cannot be seen by the patient, along with different quantitative metrics. Metrics of distortion size included the LDI, calculated as the ratio of the area or points missed by the subject and the total area explored, and is expressed as a percentage (%). Best Fit Circle Radius (BFC_Radius_) is defined as the circle that best fits to the distortion area resulting from the linear binding of all points in each meridian of the device. The higher values of LDI and BFC_Radius_ are interpreted as a lower ability to discriminate small stimuli surrounding the central source of light. Irregularity of the distortion area is derived as the deviation of the actual polygonal shape obtained from the BFC fit and is called the BFC Irregularity (BFC_Irreg_). The standard deviation of BFC_Irreg_, called BFC_SD_, measures how asymmetric is the departure of the actual distortion limits from the perfect circular shape of the BFC. Together, BFC_Irreg_ and BFC_SD_ can be interpreted as the deviation of the actual distortion from a perfectly rotational symmetric shape. The higher the value of this parameter, the larger the deviation from a circular shape, expressed in mm.

The corneal aberrations were derived from topography data using the Oculus Easygraph (Oculus, Dutenhofen, Germany) for a circular aperture of 6.0 mm. The root mean squares (RMS) for 4th- and 6th-order spherical aberration (spherical-like), third- and fifth-order horizontal and vertical coma aberration (coma-like), and fourth- and sixth-order secondary astigmatism (secondary astigmatism) were calculated.

Decimal high-contrast visual acuity and CSF were measured at distance of 5 m under photopic conditions with the LCD screen 22′′ LCD (Topcon CC-100XP, Tokyo, Japan). Frequencies tested were 1.50, 2.12, 3.00, 4.24, 6.00, 8.49, 12.00, 16.97, and 24.00 cycles per degree (cpd).

### 2.2. Statistical Analysis

Statistical analysis was conducted using SPSS software v15.0 (SPSS Inc., Chicago, IL). Descriptive statistics of the variables measured in the study were obtained. Normality of data distribution was assessed with Kolmogorov-Smirnov test. Changes in different parameters from baseline to subsequent visits were compared using ANOVA test with Bonferroni correction. Correlations between different parameters were performed using Pearson correlation. Statistical significance criterion was established at *p* < 0.05.

## 3. Results

All subjects included in the analysis showed a monocular visual of 1.0 decimals or better at the 1-month and 1-year visits.

### 3.1. Light Distortion

**Figure 1:**
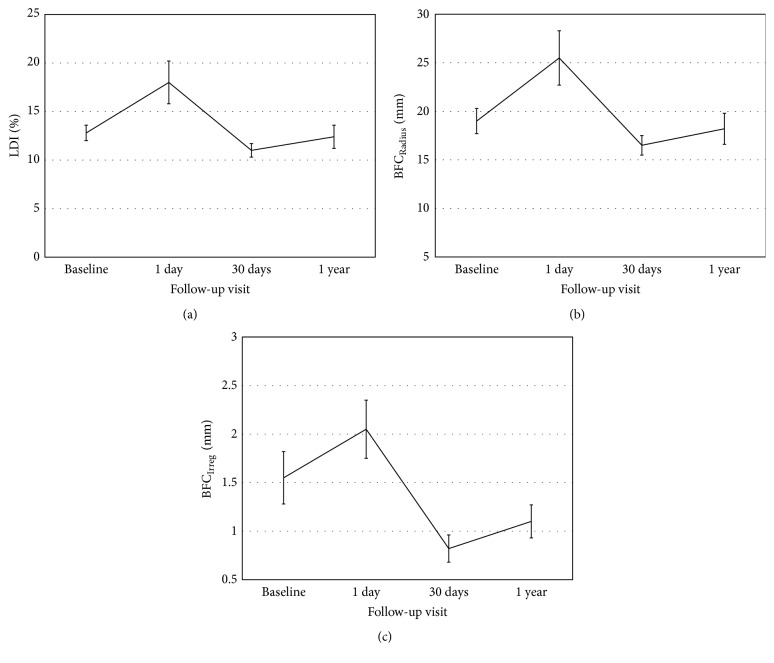
Monocular LDI (a), BFC_Radius_ (b), and BFC_Irreg_ (c) parameters. Error bars represent the standard error of the mean (SEM).

Figure 1 shows the variations of size-related (LDI and BFC_Radius_) and irregularity-related (BFC_Irreg_) parameters of monocular LD over time. BFC_Radius_ worsened after first night recovering normal values after 1 month and 1 year (ANOVA with Bonferroni post hoc correction, *p* < 0.05). BFC_Irreg_ followed a similar path with an increase after the first night and a reduction towards baseline values after 1 month (Table 2) and remaining stable after 1 year of follow-up.

While the size-related parameters of the LD recovered to baseline values, LD regularity parameters were lower after 1 month compared to baseline.

### 3.2. Corneal Aberrations

**Figure 2:**
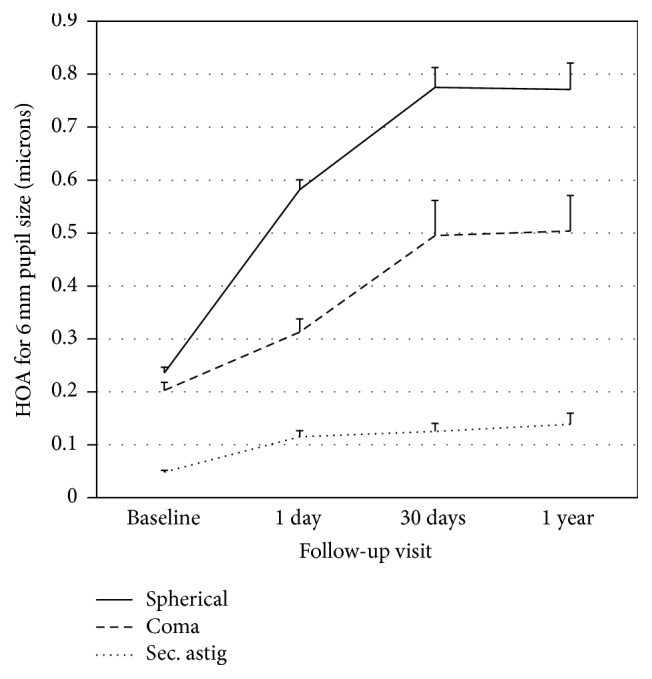
Optical quality of the corneal front surface for 6 mm pupil size represented by the root mean square (RMS) of spherical-like aberrations, coma-like aberrations, and secondary astigmatism. Error bars represent the standard error of the mean (SEM).

Figure 2 shows the variations of optical quality of the anterior corneal surface over time.  Table 3 summarizes the average differences and statistical significance for those parameters where the RMS presented a significant change between two visits as analyzed with ANOVA with Bonferroni post hoc correction. Spherical-like RMS underwent a statistically significant increase after the first night of treatment and worsened further up until the 1-month visit compared to baseline (*p* < 0.05). Coma-like RMS, as well as to a less extent secondary astigmatism RMS, showed a statistically significant increase up until the first month visit, remaining stable at the 1 year visit.

### 3.3. Contrast Sensitivity Function

**Figure 3:**
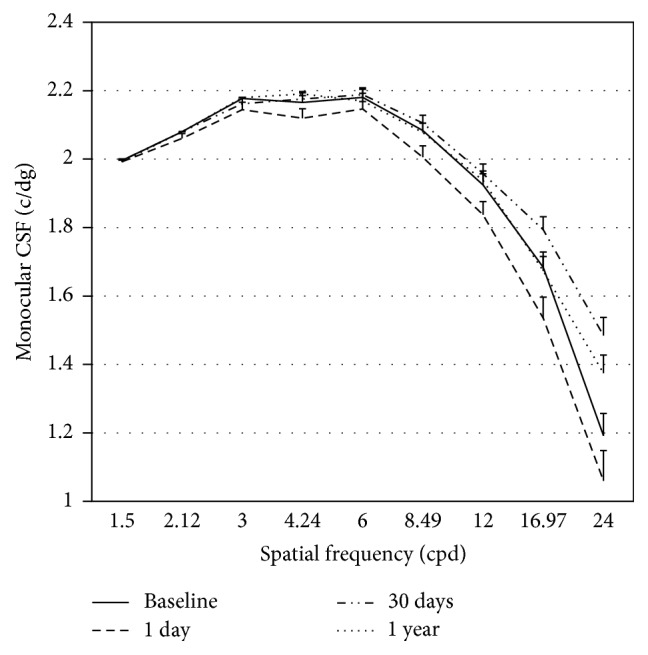
Monocular CSF. Error bars represent the standard error of the mean (SEM).

Figure 3 shows the variations in CSF between baseline and the 3 follow-up visits after 1 day, 1 month, and 1 year of treatment.  Table 4 presents the average difference and the statistical significance for those spatial frequencies showing statistically significant changes over the study period. Bonferroni post hoc correction showed that there was only a significant decrease in CS for frequencies 3.00 and 8.49 cpd from baseline on day 1. Furthermore, significant changes from day 1 to 1 month were also observed for 4.24, 8.49, 16.97, and 24.00 cpd spatial frequencies. Finally, spatial frequencies of 3.00, 4.24, and 24.00 cpd presented statistically significant changes between 1 day and 1 year.

### 3.4. Correlations

We observed that there was a statistically significant correlation between monocular CS for medium frequencies (3.0, 4.24, and 6.00 cpd) and LD parameters at baseline (*r* ≤ −0.529, *p* < 0.001). However, after 1 year of treatment, this correlation was only statistically significant for 12 cpd spatial frequency (*r* ≤ −0.565, *p* < 0.001).

In general, HOA were not significantly correlated with LD. However, after one year of treatment, we observed that there was a statistically significant correlation between spherical-like RMS for 6 mm pupil size and irregularity of LD (*r* = −0.420, *p* < 0.05).

Regarding correlations between monocular CS and HOA, correlations were statistically significant between the spherical-like RMS for 6 mm pupil size and frequencies 4.24  (*r* = 0.335, *p* < 0.05) and 12.00 cpd (*r* = 0.367, *p* < 0.05) at baseline. After 1 year of treatment, HOA were not significantly correlated with monocular CS values except for the 4.24 cpd spatial frequency against coma-like RMS (*r* = −0.397, *p* < 0.05) and secondary astigmatism RMS (*r* = −0.419, *p* < 0.05).

## 4. Discussion

With the present study we found that despite a significant and stable deterioration of visual quality, as observed in the aberrometric structure of the anterior corneal surface, visual quality measured through CSF returns to baseline values within the first month of treatment. Similarly, LD perception measured with an experimental device also returned to baseline over the first month of treatment and remained stable up to 1 year of follow-up. In OK practice, subjects occasionally report visual disturbances, even though their high-contrast visual acuity is excellent [22].

Although optical quality of the eye and quality of vision after OK have been investigated [5, 22, 23], in the present study, we evaluated HOA, CS, and adaptation to LD simultaneously in subjects undergoing OK for myopia during the first year of treatment.

Measurement of CS can provide useful information about visual function that may not be revealed by standard visual acuity testing [24, 25]. Previous studies have reported significant visual quality changes after OK with significant increase of HOA and reduction in CS depending on the amount of myopic correction [22]. In that study, the authors observed that BCVA was maintained at baseline over the follow-up period. Ocular HOA significantly increased 1 month after the procedure and remained stable thereafter. Regarding CS, there was an initial loss during overnight OK, and the loss persisted during the 1-year follow-up. Our present results do not agree with the persistent reduction in CS. Instead, we found that CS recovered to baseline values after 1 month of treatment.

Similar to the findings previously reported by us in the shorter term [14] monocular CSF experienced a significant decrease of 3.00 to 8.49 frequencies from baseline to day 1. In the present study there was a correlation between CSF and HOA at baseline and this correlation was lost after treatment onset and during the year of follow-up. On the other hand, LD parameters and HOA were not correlated during the study period, except for spherical-like RMS for 6 mm pupil size and BFC_Irreg_ after one year of treatment. In a previous work, Villa et al. reported a significant correlation between spherical-like RMS and LD [26]. Although LD and CS are recovered with respect to baseline point, there was a lack of correlation between them in the present study. This might reflect that LD itself does not in fact impair CS under the conditions of examination. It might be expected that performing CS analysis under glare conditions might result in a significant correlation between LDI and CS, but not for the conditions under which we evaluated CS in the present study. It has been surprising to find an improvement in the higher frequencies for the CSF measurement, improving from baseline to 1 year after an initial decrease. This improvement over the best corrected CSF before treatment might be related in part to some learning effects after repetitive application of the test. However, this is less likely because the tests were applied for a long period of time apart from each other.

Several studies have shown that CS significantly correlates with some abilities associated with the patient's quality of life, such as reading speed [27] or driving performance [28]. However, CS analysis is not sufficient to understand certain complaints reported by patients. This is well recognized in subjects who have undergone corneal refractive surgery and report frequent symptoms of night vision disturbances [26, 29, 30] even when high-contrast visual acuity is excellent [31–35].

In order to evaluate other aspects of the visual quality of the patient, in this longitudinal study, we investigated changes in perception of LD, changes in ocular HOA, and CSF as representative parameters of vision quality in eyes undergoing overnight OK during one year. LD analysis showed a transient increase followed by a reduction to baseline levels over the first month of treatment. Changes observed in the long-term from 1 month to 1 year are not significant. This is in agreement with the clinical observation of adaptation to the distortion effect. In a recent work we have observed that subjective reports of light distortions increase when treatment starts and decreases over the period of treatment [13].

With the present study we can conclude that adaptation phenomena observed previously in the shorter-term [14] is not expected to change in the longer term. Our observations with this experimental device are also in agreement with the time course of night vision disturbances after refractive surgery. Though OK and corneal refractive surgery are different procedures, similar time course changes are found with HOA remaining high after the procedure while the complaints and subjective perceptions of the patients seem to improve faster after the procedure [36]. Pop and Payette [30] showed a reduction in patients reporting night vision disturbances from 25% at 1-month visit to 4.7% at the 12-month visit. Our results, however, show that this reduction in night vision complaints might be faster, recovering to baseline after 1 month. This might be explained because the measurement device, such as our LD analyzer, is sensitive to more severe forms of LD phenomena that do not allow to see objects around the bright source of light. Even when the patient has recovered to a point where this kind of distortion is no longer present or is attenuated, the patient might still subjectively report it to some degree. McAlinden et al. evaluated the time course changes in the responses to the Quality of Vision (QoV) questionnaire in patients undergoing myopic and hyperopic LASEK corneal surgery [36]. This instrument accounts for frequency, severity, and bothersome of visual symptoms including haloes and starburst and we include these kinds of phenomena below the umbrella of LD phenomena. They also observed a rapid decline in the QoV score after PRK surgery with the QoV scores returning to baseline after 1 month following the treatment. At present there is no objective (and available) method to measure those photic phenomena isolated. Instruments such as subjective questionnaires do not capture these features specifically. The one that approaches such measures in a somewhat specific way is the Quality of Vision questionnaire [36]. However, this includes other aspects of subjective visual complaints and thus cannot be directly compared.

Indeed, HOA of the anterior corneal surface changed significantly in our study, in agreement with previous work [8]. In a recent paper conducted by our group, where we have evaluated HOA during the first month of OK treatment for one month, and the present results demonstrate the stability in the longer term from 1 month to 1 year of treatment [14]. The time course changes in HOA observed in the present study are in agreement with values reported by other authors [5, 22] with spherical-like and coma-like aberrations being the main contributors to the degradation of the image quality [7]. This might be justified by the fact that 3rd- (coma-like) and 4th-order (spherical-like) aberrations are more directly related to the creation and consolidation of the treatment zone, while secondary astigmatism affects more peripheral areas of the cornea presumably more subjected to day-to-day variations as a consequence of slight decentrations or pressure changes at the edge of the transition zone of the lens.

The present study has several limitations. We have not measured whole-eye HOA that could correlate better with the visual functions that we aim to evaluate. However, the main changes in aberrations in our study are induced in the front corneal surface, and it is expected that the whole eye aberrations would suffer similar changes. We cannot ensure that the LD parameter reflects specifically the haloes, ghost images, starburst, and glare reported subjectively by the patient. However, we have reported in a recent study that the system is sensitive to the photic phenomena induced when we artificially incorporate different amounts and sign of HOA [18]. Although we cannot quantify each one of the photic phenomena, we consider that such effects are well reflected in the short-term measurements in this study and that adaptation takes place to reduce the size of the LD overtime in the middle [14] and longer term (present study), in agreement with transient subjective complaints expressed by the patient in the clinical setting [13]. On the other side, the lack of correlation between LD and CS values might be related to the fact that we did not use a glare source while measuring the CSF. Compared to our previous study [14] we did not measure binocular functions of LD and CS in the present study. This does not reflect the actual viewing conditions of the patients, but we aimed to maximize the potential changes to be observed and we know that they would be more likely detected under monocular conditions, as binocular summation will tend to improve the results of LD [14, 37] and CS [38]. We also aimed to explore the correlations with HOA specific to each eye, which forced us to do follow a monocular analysis in this part of the study. Other factors that might limit the correlation between optical quality (HOA) and visual quality (LD and CS) parameters include the fact that we cannot ensure that the pupil area in each condition is exactly the same. Furthermore, neural adaptation mechanisms might play a significant role in adaptation to a deteriorated optical quality while preserving a good visual function. This ability to adaptation has been reported previously in the presence of HOA in human eyes [13, 39–41].

In summary, we have observed that the previously reported adaptation phenomena to increase HOA are maintained in the longer term, at least as far as it concerns the LD and CSF. To our knowledge this is the first study addressing the long-term changes in the optical quality of the anterior corneal surface with orthokeratology treatment and the potential impact on visual quality measured through monocular CSF and LD measurement.

## Figures and Tables

**Table 1:** Demographic, refractive, and keratometric data of subjects (mean ± SD) and range [minimum and maximum].

AGE	21.40 ± 8 years [18 to 42]
SAMPLE (male/female ratio)	20 subjects (4/16)
M (baseline)	−2.19 ± 0.97 D [−1.00 to −4.75]
J0 (baseline)	−0.04 ± 0.21 D [−0.50 to 0.46]
J45 (baseline)	0.01 ± 0.12 D [−0.29 to 0.36]
Flattest keratometric radius	7.74 ± 0.29 mm [7.20 to 8.64]
Steepest keratometric radius	7.63 ± 0.30 mm [7.06 to 8.63]
Decimal VA (monocular)	1.18 ± 0.09 [1.00 to 1.50]

**Table 2:** Statistically significant changes in parameters of monocular LD overtime (mean difference ± SEM).

		Δ ± SEM	Sig.
LDI	1 day versus 1 month	0.69 ± 0.19	0.007
BFC_Radius_	1 day versus 1 month	1.80 ± 0.47	0.005
BFC_Irreg_	1 day versus 1 month	1.00 ± 0.25	0.003

**Table 3:** Changes in HOA between baseline and follow-up visits (mean difference ± SEM) for RMS values showing statistically significant changes for 6 mm pupil size.

			Δ ± SEM	Sig.
Spherical-like	Baseline versus	Day 1	0.35 ± 0.05	<0.001
		1 month	0.54 ± 0.05	<0.001
		1 year	0.54 ± 0.05	<0.001
	Day 1 versus	1 month	0.19 ± 0.05	0.006
	Day 1 versus	1 year	0.19 ± 0.05	0.008

Coma-like	Baseline versus	1 month	0.29 ± 0.08	0.009
		1 year	0.30 ± 0.08	0.006

Secondary astigmatism	Baseline versus	1 month	0.08 ± 0.02	0.022
		1 year	0.09 ± 0.02	0.002

**Table 4:** Changes in CS between baseline and follow-up visits (mean difference ± SEM) for those frequencies showing significant changes between visits.

			Δ ± SEM	Sig.
Log CS 3.00 cpd	Day 1 versus	Baseline	0.04 ± 0.01	0.006
		1 year	0.05 ± 0.01	0.044

Log CS 4.24 cpd	Day 1 versus	1 month	0.07 ± 0.02	0.042
		1 year	0.08 ± 0.02	0.022

Log CS 8.49 cpd	Day 1 versus	Baseline	0.09 ± 0.03	0.017
		1 month	0.13 ± 0.03	0.001

Log CS 16.97 cpd	Day 1 versus	1 month	0.25 ± 0.06	0.004

Log CS 24.00 cpd	Baseline versus	1 month	0.24 ± 0.07	0.041
	Day 1 versus	1 month	0.41 ± 0.08	0.000
		1 year	0.35 ± 0.09	0.005
